# Association between Postmenopausal Osteoporosis and Experimental Periodontitis

**DOI:** 10.1155/2014/316134

**Published:** 2014-02-10

**Authors:** Kai Luo, Souzhi Ma, Jianbin Guo, Yongling Huang, Fuhua Yan, Yin Xiao

**Affiliations:** ^1^School and Hospital of Stomatology, Fujian Medical University, 246 Yangqiao Zhong Road, Fuzhou, Fujian 350002, China; ^2^Institute and Hospital of Stomatology, Nanjing University Medical School, 30 Zhongyang Road, Nanjing, Jiangsu 210008, China; ^3^Institute of Health and Biomedical Innovation, Queensland University of Technology, Brisbane, QLD 4059, Australia

## Abstract

To investigate the correlation between postmenopausal osteoporosis (PMO) and the pathogenesis of periodontitis, ovariectomized rats were generated and the experimental periodontitis was induced using a silk ligature. The inflammatory factors and bone metabolic markers were measured in the serum and periodontal tissues of ovariectomized rats using an automatic chemistry analyzer, enzyme-linked immunosorbent assays, and immunohistochemistry. The bone mineral density of whole body, pelvis, and spine was analyzed using dual-energy X-ray absorptiometry and image analysis. All data were analyzed using SPSS 13.0 statistical software. It was found that ovariectomy could upregulate the expression of interleukin- (IL-)6, the receptor activator of nuclear factor-**κ**B ligand (RANKL), and osteoprotegerin (OPG) and downregulate IL-10 expression in periodontal tissues, which resulted in progressive alveolar bone loss in experimental periodontitis. This study indicates that changes of cytokines and bone turnover markers in the periodontal tissues of ovariectomized rats contribute to the damage of periodontal tissues.

## 1. Introduction

Periodontitis is a chronic and destructive disease of the periodontium caused by various factors such as periodontal pathogens; it is commonly characterized by inflammation of periodontal tissue and alveolar bone absorption [1].

Osteoporosis is a systematic, bone metabolism-related disease with bone loss and destruction of fine bone structures that increases bone fragility and the risk of fracture. It is extremely common among the elderly, particularly in postmenopausal women. Postmenopausal osteoporosis (PMO) is osteoporosis that occurs after menopause because of the decrease in estrogen. The ovariectomized rat is a useful animal model for the study of osteoporotic bone related diseases caused by estrogen deficiency. This model exhibits a progressive loss of bone matrix through a process similar to what occurs during PMO [2, 3].

Epidemiologic research shows that chronic periodontitis is related to osteoporosis. Several studies have already indicated that insufficient estrogen is closely related to periodontitis and osteoporosis. Recently, an increasing number of researchers suggest that PMO promotes periodontitis [4–6]. It has been demonstrated that periodontal bacteria promote the alveolar bone loss in periodontitis. The invasion of periodontal bacteria may reduce bone density and enhance osteoclastic activity by releasing toxins and/or inflammatory cytokines [1]. These cytokines believed to be involved in alveolar bone remodeling are also highly expressed in PMO [7]. Since estrogen inhibits the expression of the inflammatory cytokines, it might be that larger amounts of these cytokines are presented in an inflammatory alveolar bone with estrogen deficiency. Therefore, estrogen deficiency may contribute to the alveolar bone absorption in periodontal disease, either by reducing the bone mass of alveolar bone or by causing increased expression of inflammatory cytokines. However, the underlying mechanisms are still not clear.

The homeostasis of bone tissues is controlled by the dynamic balance between osteoblastic bone formation and osteoclastic bone resorption. An imbalance between these cell activities contributes to various bone metabolic diseases. Osteoclast activation and maturation are regulated by three recently discovered proteins: the receptor activator of nuclear factor-*κ*B (RANK), RANK ligand (RANKL), and osteoprotegerin (OPG) [8, 9]. The binding of RANKL to RANK on preosteoclasts initiates the differentiation and proliferation of these cells and promotes osteoclast fusion and activation. Moreover, the activation of the RANKL-RANK pathway suppresses osteoclast apoptosis, thereby increasing the number of activated osteoclasts. On the other hand, OPG inhibits this pathway by binding to RANKL. Thus, the aforementioned proteins play essential roles in the development and maintenance of bone tissues [10, 11].

During the past decade, considerable evidence suggests that estrogen prevents bone loss by blocking the production of proinflammatory cytokines, such as interleukin-1 (IL-1), IL-6, IL-10, tumor necrosis factor- (TNF-) *α* in bone marrow and bone cells [12, 13]. The main consequence of increased cytokine production in the bone microenvironment is expansion of the osteoclastic pool because of increased osteoclast formation and their extended lifespan. To investigate the potential mechanism of PMO in periodontitis initiation and progression, we investigated the correlation between PMO and the pathogenesis of periodontitis in rats under estrogen deficiency (ovariectomy). The expression of IL-1*β*, IL-6, IL-10, TNF-*α*, OPG, RANKL, and MMP-8 in periodontal tissues, with or without osteoporosis, was analyzed using enzyme-linked immunosorbent assays and immunohistochemistry.

## 2. Materials and Methods

### 2.1. Animals

All animal care and study protocols were approved by the Animal Care and Use Committee of Fujian Medical University. A total of 24 three-month-old female Sprague-Dawley (SD) rats (220–260 g) were purchased from an animal resource centre (SLAC Laboratory Animal Co. Ltd., Shanghai, China). The rats were maintained in an animal room with 12 h day-night cycles, an ambient temperature of 22 ± 2°C. Food and water were provided *ad libitum*.

### 2.2. Surgical Protocols

Anesthesia was achieved through intramuscular injection of 4 : 1 ketamine-xylazine solution at 0.15 mL/100 g body weight. Bilateral ovariectomy was performed in 12 rats as previously described [14]. Sham surgeries were performed in other 12 rats when the ovaries were exposed but not removed. The rats were randomly divided into four groups: Group I (*n* = 6), sham-operated (SHAM); Group II (*n* = 6), ovariectomy (OVX); Group III (*n* = 6), SHAM + experimental periodontitis (EP); Group IV (*n* = 6), OVX + EP. At 10 weeks after surgery, EP was induced by placing 4-0 sterile silk ligatures around the cervix of the left upper second molar in Groups III and IV.

### 2.3. Tissue and Serum Preparation

At 2 weeks after ligation, all rats were euthanized using an overdose of anesthetic. Blood samples were taken after euthanizing the rats. The serum was separated by centrifugation for 5 min at 3,000 rpm. The samples of the three molars including the left maxillary were dissected, fixed for 48 h using 4% paraformaldehyde in phosphate-buffered saline (PBS) at 4°C, decalcified, dehydrated, and then embedded in paraffin blocks.

### 2.4. Measurement of Bone Mineral Density

At 2 weeks after ligation, all rats were euthanized through an overdose of anesthetic. The dual-energy X-ray absorptiometry (DEXA) was used to measure the bone mineral density (BMD) of the whole body, the pelvis, and the spine.

### 2.5. Measurement of Alveolar Bone Loss

**Figure 1:**
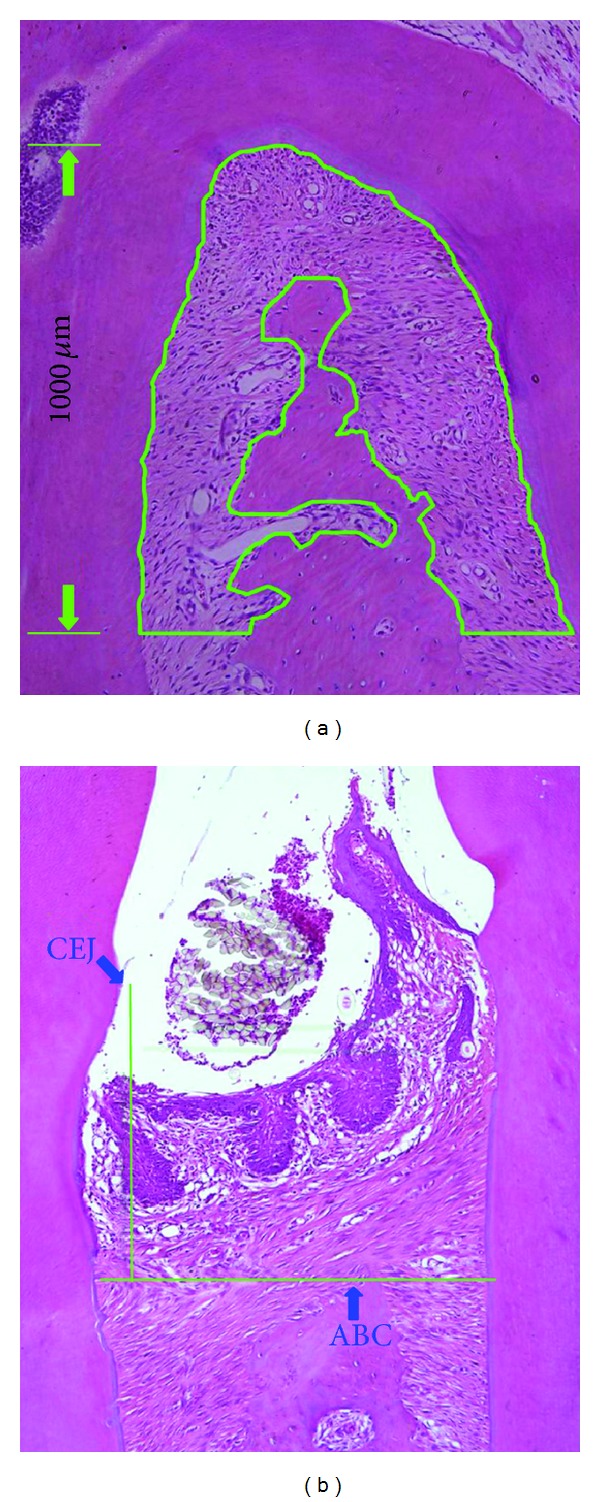
Quantitative analysis of alveolar bone loss. (a) The area enclosed by the green line represents alveolar bone absorption in upper second molar; (b) the distance from CEJ to ABC represents alveolar bone absorption. CEJ: cemento-enamel junction; ABC: alveolar bone crest.

Alveolar bone loss was evaluated using the following indices: (*A*) the area of the periodontal ligament in the root furcation of the upper second molar, with a vertical distance of 1 mm to the top of the furcation; (*B*) the distance from the cement-enamel junction (CEJ) to the alveolar bone crest (ABC) within the placement zone of the ligature and the contralateral zone.  Figure 1 shows the method of these measurement indices and the results were analyzed using Image-pro Plus 6.0 (Image-pro Plus, Media Cybernetics, Inc., USA).

### 2.6. Enzyme-Linked Immunosorbent Assays

The serum levels of BAP, TRAP 5b, IL-1*β*, TNF-*α*, and IL-6 were assayed by enzyme-linked immunosorbent assay techniques using commercial kits (R&D Systems, Minneapolis, MN, USA), according to the manufacturers' recommendations. Each serum cytokine was determined through its optical density, recorded at 450 nm using a microplate spectrophotometer.

### 2.7. Immunohistochemical Analysis

Tissue sections (5 *μ*m) were prepared for immunohistochemical study. After deparaffinization and rehydration, the sections were heated in citrate buffer using a pressure cooker to unmask the antigenicity of antigens masked by aldehyde fixation. They were then washed with PBS and treated with 3% hydrogen peroxide for 10 min to block endogenous peroxidase activity. The sections were incubated for 60 min at room temperature with primary antibodies: goat polyclonal anti-IL-6 (SC-1265; Santa Cruz Biotechnology, Inc., Santa Cruz, CA, USA), goat polyclonal anti-RANKL (SC-7628; Santa Cruz Biotechnology, Inc., Santa Cruz, CA, USA), goat polyclonal anti-OPG (SC-8468; Santa Cruz Biotechnology, Inc., Santa Cruz, CA, USA), rabbit polyclonal anti-IL-1*β* (SC-7884, Santa Cruz Biotechnology, Inc., Santa Cruz, CA, USA), rabbit polyclonal anti-TNF-*α* (SC-8301, Santa Cruz Biotechnology, Inc., Santa Cruz, CA, USA), rabbit polyclonal anti-MMP-8 (2145-1, Epitomics Inc., Epitomics, CA, USA), or rabbit polyclonal anti-IL-10 (bs-0698R, Beijing Biosynthesis Biotechnology, Beijing, China). After washing in PBS, the sections were incubated for 15 min at room temperature with one of the following secondary antibodies: HRP polymer anti-rabbit (KIT-5004, Maixin-Bio, Fuzhou, China) to detect IL-1*β*, TNF-*α*, MMP-8, and IL-10 positive cells. Samples used to detect IL-6, RANKL, and OPG were incubated for 10 min with biotinylated rabbit antigoat immunoglobulin (KIT-9709, Maixin-Bio, Fuzhou, China) and then incubated for 15 min with peroxidaseconjugated streptavidin at room temperature. After washing in PBS and visualizing using 3,3-diaminobenzidine for 5 min, all the sections were counterstained with hematoxylin for 20 s and then rinsed in running water. Finally, the sections were dehydrated in ascending concentrations of alcohol, cleared with xylene, and then mounted. Controls for the immunostaining procedures were obtained by omitting the primary antibodies or substitution with nonspecific antibodies. The sections were evaluated by a single examiner who was blinded to the treatment assignment under a microscope (1X71, Olympus Co., Tokyo, Japan) with a camera mounted on a computer. Three selected areas (50 × 50 *μ*m) in the furcation of each section were used to count positive stained cells.

### 2.8. Statistical Analysis

The quantitative data include the distance from the CEJ to the ABC, serum cytokine concentrations, the area of the periodontal ligament in the root furcation. All data were subjected to paired *t*-tests using SPSS 13.0 (SPSS, Chicago, IL, USA) statistical software, and differences were considered significant when *P* < 0.05.

## 3. Results

### 3.1. Changes in the Whole Body, the Pelvis, and the Spine BMD Levels

Compared with the SHAM group, the BMD of whole body, pelvis, and spine in the OVX group decreased significantly. Compared with the SHAM + EP group, the BMD values in the OVX + EP group also decreased significantly (*P* < 0.05).  Table 1 shows the BMD values in the four groups.

### 3.2. Histometric Results of Alveolar Bone

**Figure 2:**
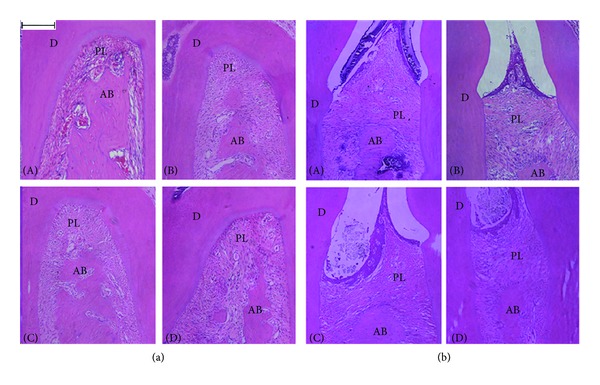
Histological aspect of upper second molar furcation area (a) and the distance from the CEJ to the ABC (b). A: SHAM; B: OVX; C: SHAM + EP; D: OVX + EP. D: dentine; periodontal ligament area; PL: alveolar bone AB: (H&E, Bar = 200 *μ*m.)

At 12 weeks after the EP, the alveolar bone loss in the root furcation and the contralateral zone increased significantly in the ovariectomized rats (Figure 2). As shown in Table 2, the area of the periodontal ligament in the root furcation in the OVX and the OVE + EP groups was significantly bigger than that in the SHAM and SHAM + EP groups, respectively. The same trend was observed in the distance from the CEJ to the ABC.

### 3.3. Detection of Serum Cytokines

Twelve weeks after the EP, the serum cytokines mentioned previously except BAP were detected by enzyme-linked immunosorbent assay techniques. The activity of BAP was measured by automatic Chemistry Analyzer. Increases of BAP, TRAP 5b, IL-1*β*, TNF-*α*, and IL-6 were found in the group OVX compared with that in the group SHAM. Similarly, the expression of BAP, TRAP5b, IL-1*β*, TNF-*α* and IL-6 were higher in the group OVX + EP compared with that in the group SHAM + EP (Table 3).

### 3.4. Immunohistochemical Expression of Cytokines

**Figure 3:**
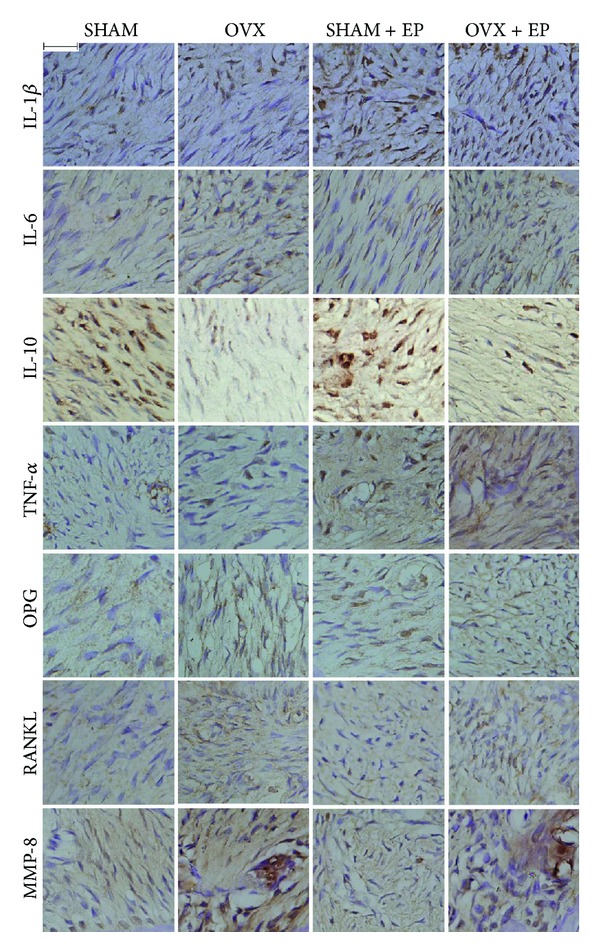
Immunohistochemical staining of the periodontal ligament in the root furcation of upper second molar in the four treatment groups. (Bar = 100 *μ*m.)

**Figure 4:**
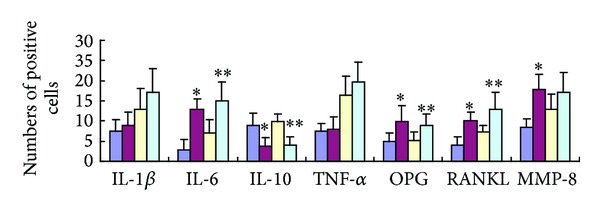
The number of cytokine-positive cells in the periodontal ligament in the root furcation of upper second molar in the four treatment groups under immunohistochemical staining. The blue, red, yellow, and green boxes represent the groups SHAM, OVX, SHAM + EP, and OVX + EP, respectively. *P < 0.05 versus SHAM rats; **P < 0.05 versus SHAM + EP rats.

Immunohistochemical staining for IL-6, OPG, RANKL, MMP-8, and IL-10 was carried out in the periodontal tissues and, as shown in Figures 3 and 4, the number of cells positive for IL-6, OPG, RANKL, and MMP-8 in the OVX and OVX + EP groups was significantly higher than that in the SHAM and SHAM + EP groups, respectively (*P* < 0.05), whereas the number of IL-10 positive cells in the OVX and OVX + EP groups was significantly lower (*P* < 0.05) compared to that in the SHAM and SHAM + EP groups, respectively. However, no changes in immunohistochemical staining for IL-1*β* and TNF-*α* were noted between these groups.

## 4. Discussion

Rats are commonly used as experimental animals because the structure of periodontal tissues and the features of PMO in rats are similar to those in humans. In addition, ligation-induced periodontal tissue inflammation is an acute periodontitis model, making it a promising model for investigating the correlation between PMO and experimental periodontitis [15–17]. Tanaka et al. found that estrogen deficiency led to alveolar bone loss, high bone turnover rates, and increased bone formation and resorption [15]. These results imply that ligation-induced periodontitis in ovariectomized rats could be an ideal model for investigating the relationship between PMO and experimental periodontitis. In present study, we demonstrated that the BMD of whole body, pelvis, and spine in ovariectomized rats decreased significantly and serum concentrations of BAP and TRAP increased after ovariectomy. BAP and TRAP are well-established metabolic markers for osteoblastic bone formation and osteoclastic bone resorption, respectively [18]. In this study, elevated serum concentrations of BAP and TRAP in ovariectomized rats implied high levels of bone resorption and formation. These typical osteoporosis profiles indicated that osteoporosis model was established successfully. Moreover, the ligation-induced experimental periodontitis exacerbated alveolar bone loss, which confirms the previous studies that postmenopause is a potential risk factor in the progression of periodontitis [4–6].

Cytokines are soluble proteins which can initiate, mediate, and control immune and inflammatory responses. It has been proposed that pro- and anti-inflammatory cytokines contribute to various bone metabolic diseases including periodontitis and postmenopausal osteoporosis (PMO) [10, 12, 19–21]. Among the proinflammatory, the IL-1, IL-6, and TNF-*α* have been reported to present fundamental role in periodontal bone destruction [22]. In present study, we found that the serum concentrations of IL-1*β*, IL-6, and TNF-*α* increased significantly in ovariectomized rats. Elevated proinflammatory cytokines in the periodontal microenvironment increase the number of osteoclasts by promoting osteoclast precursors to differentiate into osteoclasts and extending the lifespan of osteoclasts [12, 23]. Estrogen blocks bone loss by blocking the production of proinflammatory cytokines in the bone marrow, bone cells, and periodontal ligaments. IL-1*β* and TNF-*α* are potent promoters of bone resorption and inhibitors of bone formation, and IL-6 promotes the differentiation of osteoclast precursors into osteoclast and MMP production [16, 24]. The present study showed that the serums IL-6, IL-1*β*, and TNF-*α* concentration increased significantly, but IL-1*β* and TNF-*α* in periodontal tissue were not significantly changed, which was confirmed by immunohistochemical staining. IL-1*β* and TNF-*α* are upstream cytokines that are key factors that induce the production and secretion of downstream cytokines, and their slight upregulation leads to significantly higher expression of downstream cytokines such as IL-6. On the other hand, the lipopolysaccharide (LPS) produced by normal oral flora promotes the production of proinflammatory cytokines in periodontal ligament cells, endothelial cells, monocytes, and macrophages. Therefore, estrogen deficiency upregulates the proinflammatory cytokines produced by host cells after ovariectomy.

Maintaining the balance of proinflammatory and anti-inflammatory cytokines in the body is one of the manifestations of self-regulation [25]. Proinflammatory cytokines and anti-inflammatory cytokines mediate the regulation of periodontal tissues by estrogen. Our data showed that ovariectomy decreased the IL-10 levels in periodontal tissues, which could increase the alveolar bone loss. As far as we know, this is the first study to show IL-10 expression in periodontal tissue in an animal osteoporosis model. IL-10 has been identified as an anti-inflammatory cytokine and a B cell proliferation factor, having protecting effects on periodontal tissues destruction [26]. This interleukin is critical in the initiation and progression of periodontal inflammatory [27, 28]. Clinical researches have shown that IL-10 levels in GCF are lower in periodontitis sites, whereas the expression in healthy sites is higher [29]. Furthermore, alveolar bone loss is reportedly significant in IL-10 knockout mice [30, 31]. IL-10 has been shown to exert an inhibitory on alveolar bone resorption partly through the downregulation of the expression of IL-1*β*, IL-6, and TNF-*α* [32–35]. In addition, IL-10 is also able to inhibit MMPs and RANKL expression and concomitantly induces the production of their respective inhibitors TIMPs and OPG, reinforcing its potential protective role in periodontal destruction [35].

Receptor activators of nuclear factor-*κ*B (RANK), RANK ligand (RANKL), and osteoprotegerin (OPG) are the major regulatory proteins in osteoclastogenesis [8–11]. RANK/RANKL interactions have been shown to activate the proliferation, differentiation, multinucleation, and survival of osteoclasts. These stimulatory effects on bone resorption can be prevented by OPG, a soluble neutralizing receptor for RANKL [11]. It is well known that OPG and RANKL are essential for regulating osteoclast differentiation, maturation, and lifespan, as well as bone resorption. They play an important role in physiologic bone reconstruction and in the pathologic processes of bone loss, such as osteoporosis and periodontal diseases [36]. Studies on RANKL and OPG expression demonstrated higher RANKL and lower OPG expression levels in periodontitis, compared to healthy gingival tissue, in line with the biological mechanisms of these molecules in bone remodelling [37, 38]. Further on, the effects of OPG on periodontal bone resorption were tested in experimental periodontitis model. In this periodontitis model, coadministration of OPG reduced alveolar bone resorption and osteoclast formation on the bone surface [39]. In present study, we observed that the expression of OPG and RANKL increased after ovariectomy, which suggests that bone turnover rate in local bone tissues increased. In addition, RANKL increased more significantly than OPG, indicating that ovariectomy increases alveolar bone resorption in the root furcation area.

IL-1, IL-6, IL-10, and TNF-*α* regulate the expression of OPG, RANKL, and MMP-8 in periodontal tissues [10]. It has been reported that the regulation of LPS-induced RANKL expression by estrogen probably occurs by inhibiting the upregulation of upstream proinflammatory cytokines such as IL1*β*, IL-6, and TNF-*α*, whereas the regulation of OPG by estrogen is unrelated to upstream proinflammatory cytokines [24, 34]. Whether changes in OPG, RANKL, and MMP-8 in periodontal tissues are attributed indirectly to the changes in IL1*β*, IL-6, and TNF-*α* or due to the reductions in estrogen is still unclear and further studies are required to better understand the potential mechanisms of estrogen deficiency induced periodontal destruction.

In conclusion, ovariectomy promotes alveolar bone resorption in rats with experimental periodontitis and the possible underlying mechanism may be due to the decreased IL-10 and increased IL-6, OPG, and RANKL in ovariectomized periodontal tissues.

## Figures and Tables

**Table 1:** BMD values of whole body, pelvis and spine in four groups.

Groups	Whole body (g/cm^2^)	Pelvis (g/cm^2^)	Spine (g/cm^2^)
SHAM	0.173 ± 0.005	0.170 ± 0.004	0.158 ± 0.005
OVX	0.155 ± 0.008*	0.154 ± 0.008*	0.143 ± 0.008*
SHAM + EP	0.170 ± 0.005	0.168 ± 0.005	0.159 ± 0.006
OVX + EP	0.157 ± 0.010**	0.153 ± 0.011**	0.138 ± 0.008**

Values are expressed as means ± SEM; *n* = 6.

*P < 0.05 versus SHAM rats, **P < 0.05 versus SHAM + EP rats.

**Table 2:** Comparison of alveolar bone loss in different groups.

Groups	Area of the periodontal ligament in the root furcation (mm^2^)	Distance from the CEJ to the ABC (mm)
SHAM	0.25 ± 0.05	0.46 ± 0.03
OVX	0.33 ± 0.07*	0.69 ± 0.06*
SHAM + EP	0.35 ± 0.10	0.71 ± 0.06
OVX + EP	0.49 ± 0.12**	0.82 ± 0.07**

Values are expressed as means ± SEM; *n* = 6.

*P < 0.05 versus SHAM rats; **P < 0.05 versus SHAM + EP rats.

**Table 3:** Detection of serum cytokines in four groups of rats.

Groups	BAP (U/L)	TRAP 5b (pg/L)	IL-1*β* (ng/L)	TNF-*α* (ng/L)	IL-6 (ng/L)
SHAM	54.8 ± 7.0	1899.2 ± 256.7	22.1 ± 4.4	229.5 ± 52.2	63.8 ± 10.0
OVX	72.5 ± 8.8*	2245.3 ± 350.3*	39.0 ± 6.7*	293.3 ± 37.8*	146.0 ± 17.4*
SHAM + EP	53.8 ± 4.2	1849.0 ± 242.6	27.3 ± 5.0	236.7 ± 55.4	77.4 ± 9.6
OVX + EP	74.7 ± 8.7**	2298.8 ± 299.1**	36.9 ± 5.2**	302.8 ± 36.8**	140.5 ± 13.23**

Values are expressed as means ± SEM; *n* = 6.

*P < 0.05 versus SHAM rats; **P < 0.05 versus SHAM + EP rats.
